# Assessing the prevalence of urogenital schistosomaisis and transmission risk factors amongst school-aged children around Mapé dam ecological suburbs in Malantouen district, Cameroon

**DOI:** 10.1186/s40249-017-0257-7

**Published:** 2017-03-06

**Authors:** Adeline P. Mewabo, Roger S. Moyou, Lysette E. Kouemeni, Jeanne Y. Ngogang, Lazare Kaptue, Ernest Tambo

**Affiliations:** 1 Department Biochemistry, Higher Institute of Health Sciences, Université des Montagnes, Bangangté, Cameroon; 2 Institut de Recherches Médicales et d’Etudes des Plantes Médicinale-Centre de Recherches Médicales (IMPM-CRM), Yaoundé, Cameroon; 3 Africa Disease Intelligence and Surveillance, Communication and Response (Africa DISCoR) Institute, Yaoundé, Cameroon

**Keywords:** Urogenital schistosomiasis, Pupils, *Schistosoma haematobium*, Prevalence, Risk factors, Mapé dam

## Abstract

**Background:**

Urogenital schistosomiasis is a parasitic infection of public health importance that affects over 112 million people worldwide. The study aimed at assessing the urogenital schistosomiasis prevalence and risk factors of transmission around Mape dam suburds in Malantouen district, West, Cameroon.

**Methods:**

The study was conducted using semi-structured pretested questionnaires to collect socio-demographic and ecological data. Urine samples were also collected and used to confirm the prevalence of schistosomiasis in consented school-aged children in four primary schools between March – July 2014. Snails’ samples around the dam surburbs were also collected for taxonomy characterization and species identification. Data were compiled and quality control assessed and analysed using SPSS version 17 and Epiinfo data 3.1. *P* < 0.05 was considered statistical significance.

**Results:**

Questionnaires were administered to 229 pupils, with gender ratio of 1.04 (m/f). The prevalence of schistosomiasis haematobium was 16.6%. Mambonko school site, which is the closest to the dam suburbs, registered the greatest prevalence rate of 40%. The age group beween 10–13 years was the most infected (18.3%) and boys were more infested than girls (21.0% vs. 15.5%). Haematuria, urination pain, school absentiesm and poor performance were the major recorded complications in 39.5 and 26.3% males to female respectively. Infection rate gender disparity documented is still poorly understood and *Bulinus truncatus* collected from Mambonko suburb as potential snail intermediate host requires further studies.

**Conclusions:**

Authors advocated that schools and dam suburds sustained and innovative community-based surveillance and response targeted interventions implementation are needed to inform and support decision-making policy, but also in improving effective contextual behavioural communication changes and MDA improved uptake measures on national schistosomiasis control and elimination in Cameroon.

**Electronic supplementary material:**

The online version of this article (doi:10.1186/s40249-017-0257-7) contains supplementary material, which is available to authorized users.

## Multilingual abstracts

Please see Additional file 1 for translation of the abstract into the five official working languages of the United Nations.

## Background

Globally, Schistosomiasis is reported in 93 countries, accounts for more than 600 million vulnerable individuals with about 200 million infected people [1]. In tropics and sub-tropics, human and water contacts can be potential risk factor of schistosomiasis. Fresh waters, natural and artificial dams are areas where schistosomiasis infection and transmission dynamic take place [2]. The persistence human infection has been directly linked to contact with fresh water infested with snail intermediate host during fishing and swimming in ponds or dam water, and increasing contact with agricultural and irrigation contaminated water systems [2, 3]. It is documented that six schistosomes species are responsible for human schistosomiasis, but there are more commonly reported in literature namely (*Schistosoma*) *S. haematobium, S. mansoni* and *S. intercalatum* in Africa and particularly in Cameroun [4]. Globally, *S. haematobium* is the most prevalent species and accounts for about 112 million vulnerable populations and 80 million cases and 150 000 deaths annually. More than 85% of infested populations are severe and mainly found in sub-Saharan Africa, where more than 20 million suffered from a severe form of schistosomiasis complications, resulting to about 200 000 deaths annually [3, 4]. The socioeconomic consequences on developing countries is enormous, especially in Africa where it constitutes a major public health burden in riskiest children group impeding school attendance, absenteism illed-health and weak memory, poor performance and productivity, disability and death [1, 4, 5].

In Cameroun, more than 5 million people are at risk of *schistosome* infection, estimated 2 million are infected and mainly children/pupils between the ages of 6–15 years old made up the most vulnerable groups, followed by fishersmen/women and farmers [5, 6]. Historically, between 1949–1951, the two first sites of *S.* *haematobium* were reported by field workers in fishermen in BarombiMbo dam in kumba in South-Western region of Cameroon, where the prevalence was 100%. By 1960s, the tier site was reported in Loum, later in Nothern and Western areas of Cameroon in 1981 (Kekem) [7, 8]. *S. mansoni* was documented in fish ponds around olézoa in Yaoundé, in 1978 southern part around 60 km from Nkolmébanga near Sa’a closer to Sanaga river [2, 6, 9]. In 1981, the same species (*S. mansoni)* was reported in Northeners in Cameroun with a prevalence ranging from 4.9% (Koza) to 52.2% (Dougué) [10, 11]. *S. intercalatum* was reported on 67 patients in nursery school in Eséka in Cameroon in 1966, and was later reported in Obala, Mbalmayo, Edéa, Bokito and Yaoundé settings. Yaoundé, the capital reported very high prevalence rate due to inadequacies in WASH (water, sanitation and hygiene) programs implementation in urban/semi-urban settings, water scarcity and infestation in built fish ponds in Mélen, Obili et Olézoa districts with an overall prevelance of (24.3%) [12]. Recently, *S. haematobium* is unevenly distributed, more prevalent in unstable northern regions and refugees camps around the borders areas with Nigeria and central Africa Republic compared to Western, South-West and littotal provinces of Cameroon [12, 8].

Very few reports, data and records are available on the urogenital schistosomiasis in Western region of Cameroon. It documented that schistosomiasis epidemics occurred in schools environment in Magba district, Noun department in 2012, and mass praziquantel administration (MPA) has been implemented in the locality [2, 5, 6]. Since then, little consistent and comprehensive efforts in gathering quality data and information has been documented in understanding, the ecological and epidemiogical determinants of persistent schistosomiasis endemicity and flash epidemics. As Mape dam and linked fresh water rivers suburds are still inhabited by low resource populations of farmers and herbers that appear favorable conditions to parasite and hosts abundance and competence from Western to Nothern regions in Cameroon [2, 5, 9].

This study aimed at determining the prevalence of urogenital schistosomiasis and epidemio-ecological risk factors indices in school-aged children attending public schools and residing around Mape dam suburbs, in Malantouen health district in West province, Cameroon.

## Methods

### Study site

**Fig. 1:**
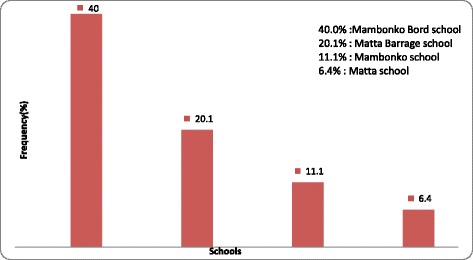
Prevelance of urogenital schistomosomaisis in school-aged children from Mape dam schools suburds, Malantouen district, West cameroon

The school-based study was conducted around the Mape dam, built in July 1987, with a maximum water level of 715 m. It covers an area of about 550 km from Adamaoua, Magba-West and North-West area in Malentouen health district. Magba is one of the nine districts, more than twenty ethnic groups (Bamon, kotoko, Bayou…) and located in Noun department in West, Cameroon. With an estimated population of 35 628 and density of 30 inhabitants per km, it is located in 5 °N and 6 °N latitude and 11 °E to 12 °E longitude. The equator climate is made up of 2 seasons: a short dry season (November to March), with temperature ranging from 30–35 °C and a longer raining season (April to October), with tempearure ranged 27–28 °C. The vegetation is dense savana, often mouldy. Agriculture and fishing have been common practice and account for 60–70% of economy and wealth source. Within structured institutional, administrative and traditional systems, schools are located in Matta and Magba villages in Malantouen district. The prevelance of urogenital schistomosomaisis was documented in school-aged children from Mape dam schools suburds, Malantouen distric (Fig. 1).

### Study duration and target population

The study was conducted within the maximum of two kilometers from the Mape dam in the health district of Malantouen in Western province of Cameroon. Stratified random sampling was deployed to select four public primary schools at varied distance from the dam namely Mambonkobord, Matta Barrage, Mambonko and Matta located at about 200 m (m), 800 m, 1 km and 2 km respectively. It covered a period of March to July 2014 and total of 229 pupils were randomly selected and enrolled according to class and school locality where pupils’ urine samples and data were collected.

### Inclusion criteria

Primary schools pupils in either classes 2, 3, 4 and 5 residing within 2 km from the Mape dam and have obtained an informed consent from the parents or legal tutor/guardian and no access to Praziquantel or other antihelmintic agents for the last 2 months were included in the study. A registered pupil in any of the selected four schools that was diagnosed with urogenital schistosomiasis was treated with praziquanted based on infected pupil body weight (Kg) and follow up for 2 months.

### Sample collection and processing

Community engagement in the selected districts were conducted with the support of local traditional, administrative and environmental sanitation authorities as well as pupils and their parents around Mape dam prior to the commencement of the study. Thereafter, further investigation to map and define schools proxy to Mape dam, water ponds and water-needs related activities in the selected villages. A pre-planned field direct physical snail samples search was perfomed using metallic tool around Mape dam and mapped neigbouring fresh river sources including aquatic plants, agricultural and fishing activities by trained staff. Snail samples were collected and placed in adequate container with fesh water, and transported to the laboratory at the Medical and Plants Research Institute (IMPM), Cameroon, where taxonomic characterization were performed.

Pupils were once again sensitized and educated on schistosomiasis prevention and control prior informed consent and enrollment. Each consented pupil received a pre-labeled contained for urine sample collected after a mild physical exercice. All data and information from pupils; and potential risk factos of urogenital schistosomiasis in the locality, clinical manifestations were recorded. Urine samples were preserved in formol solution (10% solution of formaldehyde in water) and transported to IMPM laboratory for further processing based on [13].

### Data analysis

Data were compiled and quality control assessed through a double blind process in SPSS version 17 and Epi info data version 3.1 and analysed. The chi-square (Khi2) statistics and Pearson tests were used to define the prevalence and statistical significance. A logistic regression was also performed to remove or reduce confounders. *P* value less than 0.05 was considered statistically significant.

## Results

A randomized and semi-stratified descriptive study was performed on pupils in four (4) public primary schools located within a sphere of 2 km of Mape dam, Malatouen health district, West Cameroon. A total 229 primary school-aged children, 7–16 years old from classes 2, 3, 4 and 5 were enrolled. Urine samples were collected from each enrolled and consented child parents, and further laboratory tested.

### General characteristics of the study

The average age of enrolled pupils was 11 ± 1.87 years old. There were 117 (51%) males and112 (49%) females, gender ratio of 1.04, based on the overall pupils populations in Malentoune district. Table 1 indicated that 120 (52.4%) of pupils have aged ranging within 10–13 years old (Table 1). More than half (61%) of pupils were schooling in public schools in Matta-dam (*P* = 0.01), followed by matta (27.1%) in Makounbo villages (Fig. 1).

**Table 1:** Age and gender stratified pupils’ population distribution in Malentouen, Cameroon

Age (years)	Gender				
	Male *n* (%)		Female *n* (%)		Total
[7–10]	23	(19.6)	24	(21.4)	47 (20.5)
[10–13]	62	(53)	58	(47.3)	120 (52.5)
[13–16]	32	(27.3)	30	(26.8)	62 (27)
Total	117	(51)	112	(49)	229 (100)

### Assessing age-stratified distribution of school-aged children population

Our results showed that the prevalence of infection was significantly associated with school-aged children residence duration in the area 5–10 years (28.9%) and 10–12 years (39.5%) (*P* = 0.55). Nonetheless, this prevalence increased with duration of stay and declined from 15 years, probably due to acquired immunity. The mean duration was 9 ± 3.02 years old. Our results showed that 38 (16.6%) of the studied pupils were infected*.* Gender- and age-adjusted schistosomiasis prevalence was recorded in 40% and was statistically significant in Mambonkobord, being the closest community to Mapé dam (*P* = 0.01). Pupils aged 10–13 years old were more infected (18.3%), with male gender (18.0%) more infested than female (15.1%) due to regular swimming, fishing and other related activities (Table 2).

**Table 2:** Prevalence *S. haematobium* based on residence duration in Malantouen District, Cameroon

Duration of residence in the site (years)	Prevalence of *S. haematobium* in urine		Total
	Yes *n* (%)	No *n* (%)	*n* (%)
1–5	6 (15.8)	22 (11.5)	28 (12.2)
5–10	11 (28.9)	60 (31.4)	71 (31)
10–12	15 (39.5)	62 (32.5)	77 (33.6)
12–15	6 (15.8)	47 (24.6)	53 (23.1)
Total	(100)	191 (100)	229 (100)

### Prevalence of schistosomiasis infection linked to water sources contact

**Fig. 2:**
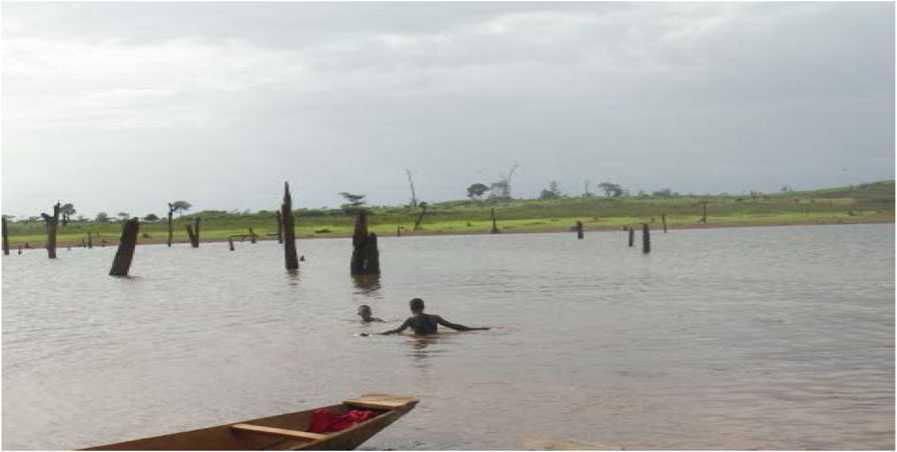
Mape Dam landscape with fishermen swimming

The prevalence of *S. haematobium* infection was 60%, from pupils that four times per week to the river/dam. 97.4% of the studied pupils were in direct contact with dam water (*P* = 0.01). A total 193 pupils (84.3%) used river and dam water for different activities including fishing and farming, laundering, bathing and cleaning of household utensils at least four times per week. The distribution of infection (89.2%) was uneven as the timing for water activities was unspecific and not regular amongst pupils contact with water sources and/or used of shared school latrines (Fig. 2).

### Clinical characteristics of studied population

Hematuria prevalence rate was reported in 39.5% and was statistically significant (*P* = 0.05); and 26.3% of pupils had recorded painful micturition (s). The prevalence of pupils that have received praziquantel treatement within three months before urine samples collections was 224 (98%) (Tables 3 & 4).

**Table 3:** Gender and age stratified distribution of studied pupils in Malentouen District, Cameroon

Age(years)	Male			Female			Total		
	Total	Positive	(%)	Total	Positive	(%)	Total	Positive	(%)
[7–10]	23	3	(13.0)	24	4	(16.6)	47	7	(14.8)
[10–13]	62	13	(21)	58	9	(15.5)	120	22	(18.3)
[13–16]	32	5	(15.6)	30	4	(13.3)	62	9	(14.5)
Total	117	21	(18)	112	17	(15.1)	229	38	(16.6)

(%)- Percentage

**Table 4:** Presenting clinical signs and symptoms amongst studied pupils in Malentouen district, Cameroon

		Presence of *S.haematobium* in the urine Percentage (%)			
		Yes *n* (%)		No *n* (%)	
Pruritis	Yes	0	(0)	0	(0)
	No	38	(100)	191	(100)
Painful micturition	Yes	10	(26.3)	8	(4.2)
	No	28	(73.7)	183	(95.8)
Macroscopic hematuria	Yes	15	(39.5)	21	(11)
	No	23	(60.5)	170	(89)

### Assessment of risk factors of *S. haematobium* infestation and schistosomiasis infection

Risk factors documented in the four schools surrounding settings included the lack of hygiene and sanitation, ignorance and lack of knowledge of the disease and the tropical ecology (relative temperature: 22–28 °C, preferable sites of the snail sample at depth of water root and stem or death leaves of 20–30 cm), that favour the development of snail intermediate host. The intermediate host of *S. haematobium* documented belongs to the family of *B*ulinidea, genius *Bulinus* made up of four subtypes namely: *africanus, tropicus*, *truncatus*, *forskali*. Also, the development of hydroelectric and agricultural policy and practice in the areas also provided another favorable biotic environment for snail development and infestation. Continuously, fishermen and farmers have been exposed to such infestation since the works involve permanent and frequent contact with water of unknown risks and determinants, requiring urgent implementation of community-based schistosomiasis risk factors surveillance and targeted interventions such as mhealth schistosomiasis strategies innovations in behavioural changes coupled with improved communities (WASH) water, sanitation and hygiene programs benefits.

## Discussions

Schistosomiasis is still a serious public health challenge in most remote settings in Africa. It is caused by the presence of *S. haematobium* in the blood vessels, and transmitted to man by an intermediate snails host in fresh water, dams and ingestion of infected food products or shared risky public schools latrines. These results showed that a total 229 pupils, aged 7–16 years old were investigated from four public primary schools located within a sphere of 2 km from Mape dam, Malantouen health district, West Cameroon. The average age of enrolled pupils was 11 ± 1.87 years old. There were 117 (51%) males versus 112 (49%) females, gender ratio of 1.04. Our results showed an average prevalence of 16.6% was infected with S. haematobium from urine samples laboratory analysis on any of the selected primary shools of the studied pupils from classes 2 to 5. These findings are consistent with similar studies reported on schools adolescents in Burkina Faso and Mali in West Africa and previously in northern dams in Cameroon [6, 8, 12, 13] Similarly, previous studies in Africa have documented the persistent of schistosmiasis in Africa. These include Congo, Cote d Iviore, Cameroon, Zimbabwe, Sudan, urogenital schistosomiasis in Pool region, on 1337 pupils with a prevalence of infestation of 15.63% carried *S. haematobium* eggs and the intermediate host characterized was *Bulinus truncatus* [7, 8, 13–15]. In Ivory Coast, a parasitological study showed that on 724 pupils reported an infestation rate of 12.6% of same species in the region of Agnéby [15, 16]. Males’ infection was higher than female directly linked to males’ behaviours, attitudes and cultural activites. In Sénégal, Niger and Mali, transversal or cross-sectional studies on risk factors and prevalence in school environment reported prevalence ranged of 30.2–72.0% and risk factors were mainly male and frequent visits and activities in fresh or dam or river water [5, 6, 9, 12, 16, 17].

Gender- and age-adjusted prevalence of schistosomiasis was 40% in school pupils and was statistically significant, mainly in Mambonkobord school, being the closest community to Mapé dam (*P* = 0.01). Pupils aged [8, 10–12] years old were more infected (18.3%), and the prevalence in male gender was more than female at (18.0) and (15.1%) respectively. The prevalence rate of *S. haematobium* infection to Mape dam was 60%, from pupils that four times per week into fresh river and dam. This result was high compared to results obtained previously in other southern and western areas in Cameroon. 37 (97.4%) were in contact with dam water (*P* = 0.01) [3, 10, 13, 16]. A total 193 pupils (84.3%) used river and dam water for bathing, fishing and cleaning of household at least 4 times per week. The distribution of infestation (89.2%) was uneven as the timing for water activities was unspecific and irregular amongst pupils [14, 15].

In terms of presence of schistosomiasis, these results are consistent with those of Deschiens in 1968 that found same three species namely *S. mansoni, S. haematobium* and *S. intercalatum*. Other epidemiological studies showed the existence of different sites that varied from regions and prevalence in men. For example the prevalence of *S. haematobium* in villages closer to SEMYI (Noulthohim, waiddoua, Madalan, Birnindel, Godjo and Maga) ranged between 44.5–61.0% and the intermediate host identified *B. truncatus* and *B. globusus* in Northern Cameroon; whereas a radio-epidemiologic study on urogenital schistosomiasis in Barombi (South–West) and recorded a prevalence higher than that documented BarombiKotto and BarombiMbo of 76 and 50% respectively [3, 5, 14, 16–18]. This intensity was not influenced by gender and age, which is opposed to our finding that showed that male were more susceptible than female. The commonest intermediate host was *B. camerunensis* and *B. truncates,* with an infestation rate 17.2%. In 2003 Njiokou [4] showed the compatibility between linked *urogenital schistosomiasis* and *S. heamatobium* with *B. truncatus and B. globusus* in Cameroon.

Recently, Boko-Haram uprising and political instability in Northern Nigeria and Central Africa Republic having huge impact (e.g., Shelther, sanitation and water scarcity, food insecurity/shortage, poor care and population displacement), and cross border movement and displacement situation continues to worsen in refugees’ camps healthcare delivery and  local inhabitants emergency disaster crisis in Northern and Eastern regions of Cameroon and requires urgent humanitarian emergency response resources and long-term capacity support.

Our finding showed that pupils age varied 7–16 years old were the dynamic group to fishing, swimming, bathing, washing and cleaning in dam water or irrigation farming activities those facilitating schistosomiasis transmission dynamics and persistence. The gender (M/F) ratio was 1.04. Our results were similar to those obtained on pupils aged 5–15 years old and ratio 1.36, but the age group [8, 10–12] years old had the high prevalence of 18.3%; while another study reported higher prevalence of 47% found in pupils between 6–10 years old [13, 14]. There was statistically significant difference between prevalence and gender (male). Pupils from Mambonkobord public school, the closest to the Mape dam were infected and this finding was consisistent with previous studies [7, 8, 15]. The reasons include the proxy and frequent contact or visit to the dam compared to those from distant villages. Since about 97.4% of infested pupils have had contact with Mape dam water, we concluded that Mape dam is the primary site of urogenital schistosomiasis incidence and prevalence in this locality [7, 15]. Pupils play an important role in the transmission dynamics which is consistent with previous findings in cameroon and other endemic areas of Africa [6, 17, 19]. This confirms that hydrolytic management constitues a significant factor *S. haematobium*. It was documented that children are in constant contact with water partially or completely during washing/bathing, swimming and cleaning households’ tools and this allow cercariae to infect them. Similarly, previous studies documented that the snail breeding sites and transmission dynamics were conditioned directly by either the abundance in *miracidium* larva in water or by human urination in aquatic environment and increasing the probability of water infestation [5, 6, 9, 12]. Swimming and bathing in stagnant water was also a positive factor in schistosomiasis emergence in domestic gardens and rice farming and consistent with previous findings [14, 18, 19]. The presence of *B. truncatus* in this site confirmed that it is favorable for the development and proliferation of snail, which is consistent with observations consistent with similar endemic *S. haematobium* studies across Africa [3, 5, 9, 12, 15, 20]. Hence, understanding  snail ecology and climatic change influence on abundance and transmission dynamics is essential in  risk mapping and evidence-based intermediate host interruption interventions.

A total of 39.5% of infection rate was linked with the duration of stay (10–11 years) in the residential site or suburd(s). This result was weak compared to previous studies [4, 7, 11, 14, 18] that reported 87.5% of patients that never left the village since they were born. However, pupils that have been lived in the locality for 12–15 years old had a lower prevalence rate of 15.8%, this can be explained by the the acquired immunity developed by these older ages through repeated exposure to infested dam water. Hematuria and painful micturition in studied school-aged children were the commonest clinical signs recorded a prevalence of 39.5 and 26.3% respectively [20, 21]. Our reported schistosomiasis prevalence of 16.6% was similar to Akouala et al. [7, 22], but low compared to Nkengazong et al. at Barombidam [23–25] in 2013 (69.17%). This study is consistent previous studies in addition to school absentiesm and poor performance [3, 5, 15, 21, 22, 26–28]. Our findings revealed that epidemio-ecological factors responsible for persistent transmission dynamics included the presence/contact with the dam, traditional fisherman culture, interation between animal-human contaminated Mape dam source of water driking, favourable subtropical climate, landscape of region, residence for at least one year and 2 km surrounding villages, and no access to Praziquantel or other antihelmintic agents for the last 2 months [2, 9, 15, 18, 22, 28, 29].

Probably, explications could be Barombi locality is known genetic diversity of urogenital schistosomiasis endemcity, intermediate host exposure and  migration/motility of the studied population crossed every day to or from their daily activities [24, 30]. The sensitive *B. truncatus* populations were sensitive to all tested *S. haematobium* and can play an important role similar to *B. globusus*in the expansion of schistosomiasis in Cameroon. Nkengazong et al.*,* in 2013 showed that in school settings *S. haematobium* in Kumba, had a prevalence of 69.17%. *Bulinus truncatus and camerunensis* were the intermediate hosts found in the locality [25, 26, 31, 32]. However, local periodic MPA has been reported and might have significantly reduced the rate of water infestation and *S. haematobium* re-infection and re-introduction or importation and prevalence amongst pupils and population round the dam suburbs including tourists [1, 2, 33, 34].

Study limitations included the study duration, biased and no define key physiochemical characteristics of mollusca sites that may require coupled with appropriate snail or cercariae breeding sites and hosts mapping. In addition,  understanding repeated MDA on schistosome resistance emergence prevention and transmission interruption as well as malacology study is crucial in determining the rate of infestation of *B.truncatus* and better interpretation of the results and outcomes. The rate of infestation of mollusca was not determined, since mosllusca were deaths before arrival at the laboratory. There will be need to associate social demographic, behavioural and epidemiological data that depends on the honest answers of the pupils.

## Conclusions

This study showed that the overall prevalence of *S. haematobium* in selected Malantouen health district sites was 16.6%. The proximity factor of Mambonkobord public school to Mape dam had the most prevalent *S. haematobium* infected pupils population and confimed clinical signs and symptoms of hematuria and micturaction pains linked to urogenital schistosomiasis were the most frequent reported in infected pupils in Malantouen health district. The identified *B. truncatus* may being probably the snail species responsible for the persistent schistosomiasis public health burden in these vulnerable communities. Strengthening evidence-based and sustainable national schistosomiasis community-based surveillance and response programs and interventions implementation should be prioritzed in improving effective contextual behavioural communication changes and improved MDA uptake strategies amongst vulnerable populations. While, leveraging on advances in mhealth approach and social media innovations networks fitness in improving sustained schistosomiasis control and elimination across endemic or epidemic prone-countries including Camerooun.

## Additional file

Additional file 1: Multilingual abstracts in in the six official working languages of the United Nations. (PDF 572 kb)

## Abbreviations

IMPM: Medical and Plants Research Institute
Kg: Weight
MPA: Mass Praziquantel Administration
