# Supplementary material for: Assessing the prevalence of urogenital schistosomaisis and transmission risk factors amongst school-aged children around Mapé dam ecological suburbs in Malantouen district, Cameroon
Source: Infect Dis Poverty. 2017 Mar 6;6:40. doi: 10.1186/s40249-017-0257-7 (PMC5338087; doi:10.1186/s40249-017-0257-7)
Supplement: Additional file 1: — Multilingual abstracts in in the six official working languages of the United Nations. (PDF 572 kb) [file 40249_2017_257_MOESM1_ESM.pdf]

Translation of the abstract into the five official working languages of the United Nations

**تقييم انتشار البلهارسيا البولية وعوامل خطر انتقالها بين الأطفال في سن المدرسة في جميع أنحاء الضواحي البيئية لسد مابي في منطقة ملنتون، الكاميرون**

أدلين ب. ميوابو، روجر س. مويو، ليزيت إ. كوئميني، جين ي. إنجوجانج، لازار كابتوي، إرنست تامبو

**ملخص**

**خلفية:** البلهارسيا البولية مرض طفيلي ذو أهمية للصحة العامة يؤثر على أكثر من 112 مليون شخص في جميع أنحاء العالم. هدفت الدراسة إلى تقييم مدى انتشار البلهارسيا البولية وعوامل خطر انتقالها في ضواحي سد مابي في حي ملنتون، الغرب، الكاميرون.

**الطرق:** أجريت الدراسة باستخدام استبيانات مسبقة الفحص شبه منظمة لجمع البيانات الاجتماعية والديموغرافية والبيئية. تم جمع عينات البول أيضا واستخدامها لتأكيد انتشار مرض البلهارسيا في الأطفال في سن المدرسة في أربع مدارس ابتدائية اشتركت في الدراسة ما بين مارس ويوليو 2014. جمعت عينات القواقع من ضواحي سد مابي أيضا لتوصيف التصنيف وتحديد الأنواع. وقد تم تجميع البيانات ومراقبة الجودة تقييمها وتحليلها باستخدام SPSS النسخة 17 و3.1 Epiinfo data واعتبرت P <0.05 دلالة إحصائية.

**النتائج:** شملت الاستبيانات 229 تلميذا، ونسبة الجنسين 1.04 (ذكر/أنثى). وبلغت نسبة انتشار مرض البلهارسيا البولية 16.6٪. مسجلة في موقع مدرسة مامبونكو، وهو الأقرب إلى ضواحي السد، وأكبر معدل انتشار 40٪. وكانت الفئة العمرية 10-13 سنة هي الأكثر إصابة (18.3٪)، وكان عدد الأولاد أكثر من البنات المصابة (21.0٪ مقابل 15.5٪). كانت البيلة الدموية، وآلام التبول، والتغيب عن المدرسة وضعف الأداء المضاعفات الرئيسية الملحوظة في 39.5٪ من بين الذكور و26.3٪ من الإناث. لا يزال التفاوت في معدل الإصابة الموثق بين الجنسين لا تزال غير مفهوم وحلزون *Bulinus truncatus* التي تم جمعه من ضاحية مامبونكو عائل وسيط المحتمل يحتاج إلى مزيد من الدراسات.

**الاستنتاجات:** يرى المؤلف أنه هناك حاجة إلى اتخاذ تدابير وقائية مبتكرة ومستديمة وعمل تدخلات هادفة لتوعية ودعم اتخاذ القرار السياسي في الخطة الوطنية لمكافحة البلهارسيا والقضاء عليها في المدارس وضواحي سد.

Translated from English version into Arabic by Mahmoud Sami, through

TRANSLATORS
WITHOUT BORDERS

**喀麦隆 Malantouen 区 Mapé 大坝生态区学龄儿童的泌尿生殖系血吸虫病患病率及其传播危险因素**

Adeline P. Mewabo, Roger S. Moyou, Lysette E. Kouemeni, Jeanne Y. Ngogang, Lazare Kaptue, Ernest Tambo

**摘要**

**引言：**泌尿生殖系血吸虫病是一种重要的公共卫生意义的寄生虫病，全球受感染人群超过 1.12 亿。本研究旨在评估喀麦隆 Malantouen 区 Mapé 大坝生态区学龄儿童泌尿生殖系血吸虫病患病率及其传播危险因素。

**方法：**本研究采用半结构化预测试问卷展开调查，收集社会人口学和生态数据。在 2014 年 3 月至 7 月，收集尿液样本，并进行检验，计算 4 所小学的学龄儿童泌尿生殖系血吸虫病的

患病率。在大坝区附近采集钉螺进行分类学特征和种类鉴定。使用 SPSS 17 版和 Epiinfo data 3.1 版进行数据整理和质量控制评估和分析。

**结果：**问卷调查了 229 名学生，男女性别比例为 1.04。埃及血吸虫病患病率为 16.6%。在距离大坝区最近的 Mambonko 学校，患病率高达 40%。感染率最高的是 10-13 岁组（18.3%），男生（21.0%）比女生（15.5%）患病率高。血尿、排尿困难、上课缺勤和学校不良表现是主要记录问题，其中男生占 39.5%，女生占 26.3%。然而，对于感染率的性别差异仍知之甚少。从 Mambonko 区采集的截形水泡螺（*Bulinus truncatus*）是否是潜在中间宿主仍需要进一步研究。

**结论：**笔者认为学校和大坝区应该维持和创新相应的预防措施，有针对性的干预措施有助于制定支持全国血吸虫病控制和消除的政策。

Translated from English version into Chinese by Yin-Long Li, edited by Pin Yang

CHINESE CENTER FOR DISEASE CONTROL AND PREVENTION

NATIONAL INSTITUTE OF PARASITIC DISEASES

## Évaluation de la prévalence la bilharziose uro-génitale et des facteurs de risque de transmission parmi des enfants en âge d'être scolarisés dans la zone environnant le barrage de Mapé dans le district de Malantouen, Cameroun

Adeline P. Mewabo, Roger S. Moyou, Lysette E. Kouemeni, Jeanne Y. Ngogang, Lazare Kaptue, Ernest Tambo

**Résumé**

**Contexte :** la bilharziose uro-génitale est une maladie parasitique de grande importance en termes de santé publique et qui affecte plus de 112 millions de personnes dans le monde entier. La présente étude a pour objectif d'évaluer la prévalence de la bilharziose uro-génitale et les facteurs de risque de transmission dans la zone environnant le barrage de Mapé dans le district de Malantouen, Ouest, Cameroun.

**Méthodes :** la présente étude a été menée à l'aide de questionnaires pré-testés et semi-structurés afin de collecter des données socio-démographiques et écologiques. Des échantillons d'urine ont aussi été collectés et utilisés afin de confirmer la prévalence de la bilharziose chez des enfants en âge d'être scolarisés et pour lesquels un consentement préalable a été recueilli au sein de quatre écoles primaires entre mars et juillet 2014. Les escargots collectés dans les environs du barrage ont aussi fait l'objet d'une taxonomie et d'une identification des espèces. Les données ont été compilées et ont fait l'objet d'un contrôle qualité avant d'être analysées à l'aide du logiciel SPSS, version 17 et Epi Info 3.1. Un $P < 0,05$ était considéré comme étant statistiquement significatif.

**Résultats :** les questionnaires ont été soumis à 229 écoliers, à un rapport garçons-filles de 1,04 (m/f). La prévalence du *Schistosomiasis Haematobium* s'élevait à 16,6 %. L'établissement scolaire de Mambonko, le plus proche du barrage, affichait le taux de prévalence le plus élevé : 40 %. Le groupe d'âge des 10-13 ans était le plus infecté (18,3 %) et les garçons présentaient un taux d'infestation plus élevé que les filles (21,0 % vs 15,5 %). L'hématurie, les douleurs à la miction, l'absentéisme scolaire et les mauvais résultats d'apprentissage étaient les principales complications consignées chez respectivement 39,5 % et 26,3 % des garçons et des filles. La disparité de genre

liée au taux d'infection est mal comprise et le rôle des escargots *Bulinus truncatus* collectés dans la banlieue de Mambonko à titre d'hôte intermédiaire potentiel doit faire l'objet d'études complémentaires.
**Conclusions :** les auteurs ont encouragé la prise de mesures préventives durables et innovantes par les écoles et dans les environs du barrage et des interventions ciblées sont requises afin de sensibiliser la population et de contribuer à une politique volontariste de lutte et d'élimination de la bilharziose à l'échelon national.

Translated from English version into French by eric ragu, through

TRANSLATORS
WITHOUT BORDERS

## Оценка заболеваемости мочеполовым шистосомозом и факторов риска передачи инфекции среди школьников в экологических районах вокруг дамбы Мапе в округе Малантуен, Камерун

Аделайн П. Мевабо (Adeline P. Mewabo), Роджер С. Мойю (Roger S. Moyou), Лизетт Е. Куемени (Lysette E. Kouemeni), Джиэнн И. Нгоганг (Jeanne Y. Ngogang), Лазар Каптю (Lazare Kaptue), Эрнест Тамбо (Ernest Tambo)

**Аннотация**
**Краткое описание.** Мочеполовой шистосомоз – это паразитарная инфекция, имеющая значение для состояния здоровья населения, так как ею поражено свыше 112 миллионов человек во всем мире. Данное исследование было направлено на оценку заболеваемости мочеполовым шистосомозом и факторов риска передачи инфекции в районах около дамбы Мапе в округе Малантуен (Malantouen), Западный регион, Камерун.
**Методы.** В ходе исследования были собраны социально-демографические и экологические данные, которые были получены с помощью полуструктурированных, предварительно протестированных опросных листов. Для определения заболеваемости шистосомозом были также собраны образцы мочи у согласившихся на обследование детей школьного возраста в четырёх начальных школах с марта по июль 2014 года. Кроме этого, в районах около дамбы были собраны образцы улиток для таксономии и определения видовой принадлежности. Данные были скомпилированы, проверены на качество и проанализированы с использованием пакетов SPSS, версия 17, и Epi Info 3.1. Уровень $P < 0,05$ считался статистически значимым.
**Результаты.** Опросные листы были выданы 229 ученикам; соотношение полов составило 1,04 (м/ж). Заболеваемость *Schistosomiasis Haematobium* была 16,6%. Наивысший показатель заболеваемости 40% был зарегистрирован в школе в Мамбонко, расположенной ближе других к районам вокруг дамбы. Возрастная группа от 10 до 13 лет была инфицирована более всего (18,3%), и мальчики были инфицированы больше, чем девочки (21,0% и 15,5% соответственно). Гематурия, боль при мочеиспускании, пропуск занятий в школе и плохая

успеваемость были основными зафиксированными осложнениями у 39,5% мальчиков и у 26,3% девочек. Подтверждённая разница в уровнях заражённости в зависимости от пола ещё плохо изучена, и собранные в районе Мамбонко улитки *Bulinus truncatus* нуждаются в дальнейшем исследовании, чтобы выяснить, являются ли они потенциальным промежуточным хозяином.

**Заключение.** Авторы выступают за проведение в школах и районах около дамбы долгосрочных и передовых профилактических мер и целевых вмешательств, направленных на информирование и поддержку ключевой программы по контролю шистосомоза и его искоренению на национальном уровне.

Translated from English version into Russian by Natalia Potashnik, through

TRANSLATORS WITHOUT BORDERS

**Evaluación de la prevalencia de esquistosomiasis urogenital y de los factores de riesgo de transmisión entre los niños en edad escolar en el área circundante a los suburbios ecológicos de la presa Mapé en el distrito de Malantouen, Camerún.**

Adeline P. Mewabo, Roger S. Moyou, Lysette E. Kouemeni, Jeanne Y. Ngogang, Lazare Kaptue, Ernest Tambo

**Resumen**

**Antecedentes:** La esquistosomiasis urogenital es una infección parasitaria de gran importancia para la salud pública que afecta a más de 112 millones de personas en todo el mundo. El objeto de este estudio es realizar una evaluación de la prevalencia de la esquistosomiasis urogenital y de los factores de riesgo de transmisión entre los niños en edad escolar en el área circundante a los suburbios de la presa Mapé en el distrito de Malantouen, Oeste, en Camerún.

**Métodos:** El estudio se ha llevado a cabo utilizando cuestionarios semiestructurados probados con antelación con la finalidad de recoger datos socio-demográficos y ecológicos. También se han recogido muestras de orina que se emplearon para confirmar la prevalencia de esquistosomiasis entre los niños en edad escolar que habían prestado su consentimiento para ello, en cuatro escuelas de enseñanza primaria entre los meses de marzo-julio de 2014. Además, se han recolectado muestras de caracoles en los alrededores de la presa para su caracterización taxonómica e identificación de especies. Se recopilaron los datos y se realizó una evaluación y análisis del control de calidad utilizando los programas de análisis de datos SPSS versión 17 y Epiinfo data 3.1. Se consideró a $P < 0{,}05$ estadísticamente significativo.

**Resultados:** Se distribuyeron los cuestionarios entre 229 alumnos, en proporción de género de 1,04 (h/m). La prevalencia de la *Schistosomiasis Haematobium* fue del 16,6%. La tasa más alta de prevalencia fue del 40% y se registró en la localización de la escuela Mambonko, que es la más cercana a los suburbios de la presa. El grupo más infectado (18,3%) era el que se encontraba entre las edades de 10-13 años y los niños estaban más infectados que las niñas (un 21,0% contra un

15,5%). Las principales complicaciones registradas fueron hematuria, dolor al orinar, absentismo escolar y bajo desempeño en proporción del 39,5% y del 26,3%, en niños y niñas, respectivamente. La disparidad de género en la tasa de infección registrada aún no se comprende cabalmente y se necesitan todavía más estudios sobre el caracol *Bulinus truncatus*, recolectado en los alrededores de Mambonko, como posible hospedador intermediario.
**Conclusiones:** Los autores sostienen que se necesitan medidas preventivas sostenidas e innovadoras e intervenciones dirigidas en las escuelas y en los alrededores de la presa para obtener información que apoye la toma de decisión para elaborar una política de control y eliminación de la esquistosomiasis a nivel nacional.

Translated from English version into Spanish by Ana Claudia Macoretta, through

TRANSLATORS
WITHOUT BORDERS
